# Refractory Chronic Eosinophilic Annular Erythema Successfully Treated with Dupilumab: A Case Report

**DOI:** 10.1159/cde/abvag023

**Published:** 2026-10-09

**Authors:** Avery H. Seward, Robert J. Pariser

**Affiliations:** a Eastern Virginia Medical School at Macon & Joan Brock Virginia Health Sciences at Old Dominion University, Norfolk, VA, USA; b Pariser Dermatology Specialists, Norfolk, VA, USA

**Keywords:** Eosinophilic annular erythema, Wells syndrome, Dupilumab

## Abstract

**Introduction:**

Eosinophilic annular erythema (EAE) is a rare eosinophilic skin condition that lacks a well-established treatment plan. It is characterized by annular, erythematous, indurated plaques which may be asymptomatic or pruritic. Histologically, EAE shares features with Wells syndrome (WS), including a dense dermal eosinophilic infiltrate, which has led to varying opinions as to whether they are distinct entities or lie on a clinical spectrum. We report a case of a woman with a well-documented 26-year history of refractory EAE that ultimately responded to dupilumab, a monoclonal antibody targeting interleukin-4 and interleukin-13.

**Case Presentation:**

The patient initially presented at age 13 with a pruritic eruption composed of arcuate plaques with raised borders, trailing scale, and prominent post-inflammatory hyperpigmentation involving the trunk and extremities. Skin biopsies performed in 2000, 2004, and 2021 demonstrated a superficial and deep perivascular and interstitial eosinophilic infiltrate. The first two biopsies showed eosinophil degranulation and flame figures, while the third remained consistent with EAE without flame figures. Laboratory evaluation revealed persistent peripheral eosinophilia (14–22%) without identifiable underlying systemic disease. Over 26 years of follow-up by a single clinician, the patient failed numerous therapies, including topical and systemic corticosteroids, calcineurin inhibitors, antihistamines, antibiotics, antifungals, imiquimod, dapsone, hydroxychloroquine, phototherapy, upadacitinib, and omalizumab, with only transient or minimal benefit. In July 2025, treatment with dupilumab was initiated using a 600 mg loading dose followed by 300 mg every other week. The patient experienced significant improvement in pruritus and marked reduction in erythema and induration of active lesions, with no new lesions developing over 9 months of follow-up.

**Conclusion:**

This case represents successful treatment with dupilumab of longstanding EAE with overlapping histologic features of WS and persistent eosinophilia and adds to the growing evidence that EAE and WS may lie on a clinical spectrum. The patient's response supports a role for IL-4/IL-13-mediated type 2 inflammation in the pathogenesis of EAE and further suggests that dupilumab may be an effective therapeutic option for refractory, chronic EAE.

## Introduction

Eosinophilic annular erythema (EAE) is a rare eosinophilic skin condition that lacks a well-established treatment plan [1, 2]. It commonly presents as annular, centrifugal, erythematous, indurated plaques on the trunk and extremities, which may be asymptomatic or pruritic [1, 3]. Wells syndrome (WS), or eosinophilic cellulitis, is a related eosinophilic dermatosis that classically presents as tender, edematous, cellulitis-like plaques that typically resolve over four to 6 weeks, with a relapsing course over months to years; histology in the subacute phase shows a dermal eosinophilic infiltrate with characteristic flame figures, while the chronic phase is more granulomatous [4, 5]. Histologically, EAE shares several features with WS, including a dense dermal eosinophilic infiltrate [2]. This overlap has led to varying opinions as to whether they are distinct conditions or exist on a clinical spectrum [3, 6, 7]. We report a case of a woman with a well-documented, 26-year history of refractory EAE which ultimately responded to dupilumab, a monoclonal antibody targeting interleukin 4 and 13.

## Case Report

In 1999, a 13-year-old girl presented with a 3-year history of a pruritic eruption. It consisted of multiple lesions characterized by arcuate, elevated borders, sometimes showing a trailing scale, with marked, trailing post-inflammatory hyperpigmentation affecting the torso, arms, legs, and back (Fig. 1). At the time, her past medical history was unremarkable, and she has since remained healthy. A punch biopsy in 2000 of an affected area on the left chest revealed moderate epidermal spongiosis and a perivascular and interstitial infiltrate of histiocytes and lymphocytes in the upper dermis along with focal, dense collections of eosinophils surrounding altered eosinophilic collagen bundles consistent with flame figures.

**Fig. 1.**
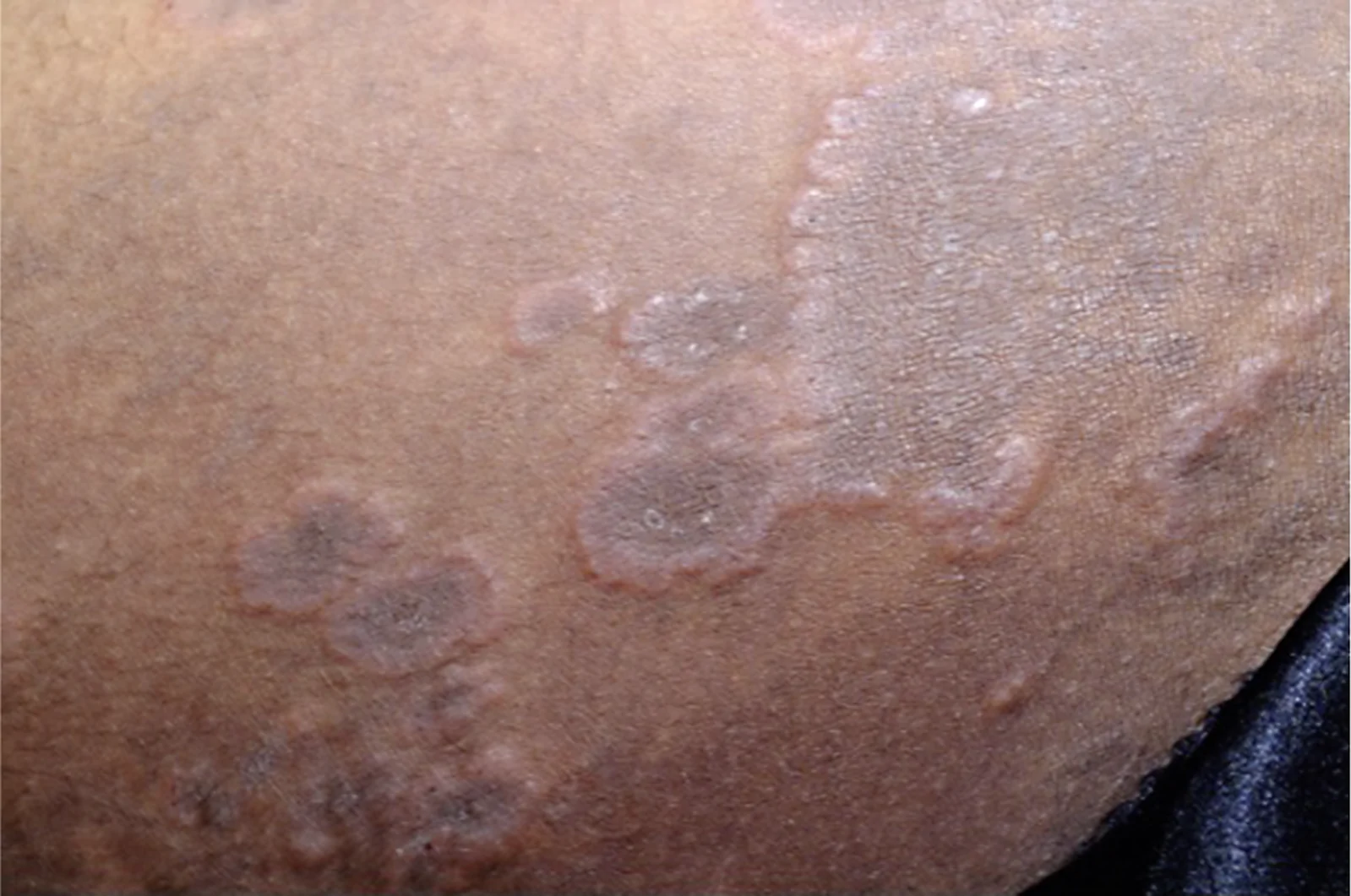
Arcuate, confluent erythematous plaques with elevated borders and central post-inflammatory hyperpigmentation on the abdomen.

A repeat biopsy in 2004 showed mild epidermal papillomatosis, a dense perivascular and interstitial eosinophilic infiltrate, and many degranulating eosinophils. PAS staining showed no organisms but highlighted prominent dermal eosinophilia and showed an early flame figure, consistent with a diagnosis of EAE. A third biopsy in 2021 again showed a dermal perivascular and interstitial infiltrate with many eosinophils, without flame figures; some eosinophils were present in the epidermis. PAS stains were negative for fungal organisms. The findings were felt to be consistent with the diagnosis of EAE.

She was followed by the same clinician (RJP) for 26 years. During this time the patient was treated with a wide range of therapies, including topical and systemic corticosteroids, tacrolimus, antihistamines, antibiotics, antifungals, imiquimod, dapsone, hydroxychloroquine, and upadacitinib without sustained benefit. More recently, omalizumab 300 mg every 4 weeks was trialed for 10 months but discontinued due to lack of efficacy. She also underwent ultraviolet A and B phototherapy in 2002 and 2017, with partial response but discontinued each time due to time constraints.

Her disease has been persistent and relapsing since presentation and has continued to occur in the absence of any identifiable underlying illness, infection, allergen, or medication. In 2004, the patient had a normal blood chemistry panel and a blood count showing an elevated eosinophil count of 14% as the sole abnormality. Subsequent laboratory evaluations have consistently demonstrated persistent eosinophilia ranging from 14 to 22%. No additional laboratory workup or evaluation for secondary causes of eosinophilia was pursued during her disease course, given her longstanding, biopsy-confirmed diagnosis of EAE and absence of clinical symptoms.

On July 15, 2025, the patient received her first 600 mg subcutaneous injection of dupilumab, followed by a 300 mg subcutaneous dose every other week. The patient has reported significant improvement in pruritus and has shown a marked reduction in induration and erythema along the annular, advancing edges of her lesions (Fig. 2). While post-inflammatory hyperpigmentation persists, no new lesions have developed over the last 9 months with 0% of body surface area affected by active disease. We plan to continue dupilumab, with periodic assessment of peripheral eosinophil counts and consideration of adjusting the dosing interval or adding a brief oral prednisone taper should flares occur. The CARE Checklist has been completed by the authors for this case report, attached as online supplementary material (for all online suppl. material, see https://doi.org/10.1159/cde/abvag023).

**Fig. 2.**
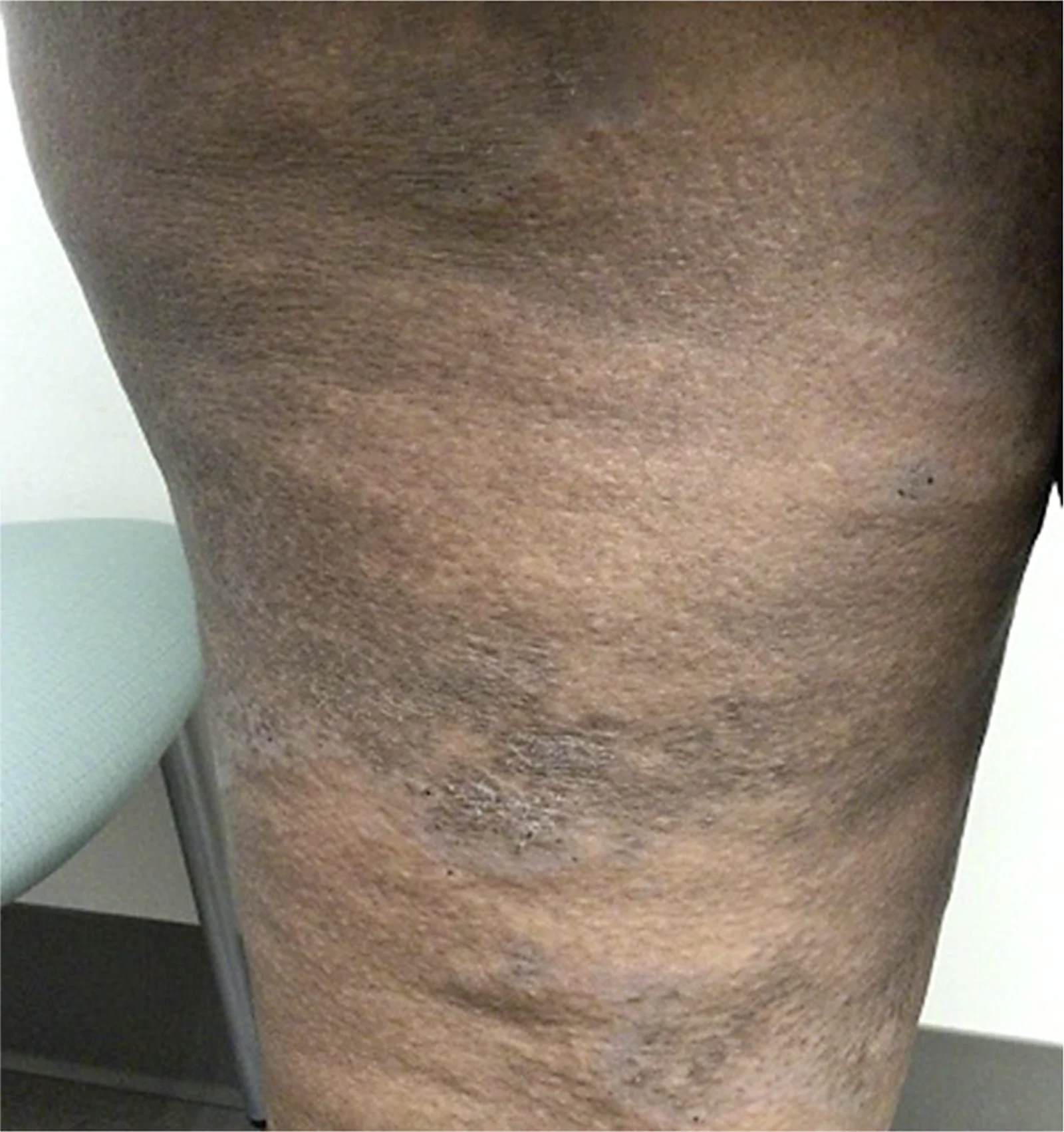
On the lateral thigh, residual post-inflammatory hyperpigmentation with marked reduction of induration and erythema along lesion periphery 7 weeks after initiation of dupilumab treatment.

## Discussion

The relationship between EAE and WS continues to be debated. EAE is characterized by annular or polycyclic, typically non-edematous centrifugal plaques with a superficial and deep perivascular and interstitial infiltrate of lymphocytes and eosinophils, but generally lacks the flame figures considered a hallmark of WS [1, 3]. In contrast, WS classically presents with tender, edematous, cellulitis-like plaques that progress through acute, subacute, and chronic phases. The subacute phase is characterized histologically by a dermal eosinophilic infiltrate with prominent flame figures, whereas chronic lesions show a more granulomatous pattern with increased histiocytes and giant cells [4, 5].

WS was an important consideration in our patient given the intermittent flame figures and persistent eosinophilia (14–22% over 26 years), but the plaques were annular and scaling rather than the edematous, cellulitis-like plaques typical of WS, favoring a diagnosis of EAE with overlapping features of WS. Our patient’s findings support the view that EAE and WS may exist on a shared spectrum, based primarily on the overlap in clinical features and histopathology and only secondarily based on response to dupilumab.

The differential diagnosis also included granuloma annulare, erythema annulare centrifugum, and subacute cutaneous lupus erythematosus. Granuloma annulare typically shows a palisaded or interstitial granulomatous infiltrate with mucin deposition rather than a predominantly eosinophilic infiltrate, and lesions are not scaly. Erythema annulare centrifugum classically shows a “trailing scale” similar to EAE but histology demonstrates a superficial or deep perivascular lymphocytic infiltrate without significant tissue eosinophilia. Subacute cutaneous lupus erythematosus presents as annular or papulosquamous erythematous plaques with histology showing an interface dermatitis with a deep perivascular and periadnexal lymphocytic infiltrate with mucin, and without flame figures.

Several case series have suggested that EAE and WS exist along a shared spectrum. El-Khalawany et al. [6] followed 10 patients with EAE and concluded that EAE may represent a variant of WS, characterized by a chronic course, treatment resistance, and high relapse rate. They noted that flame figures were present but limited to well-developed, longstanding lesions. Rongioletti et al. [7] similarly described EAE as part of the clinical and histopathologic spectrum of WS rather than a separate condition. Recent multicenter case series have added to this debate. Corio et al. [8] described four patients with EAE without flame figures on histology, while Cohen Sabban et al. [9] reported four cases lacking flame figures and favored classifying EAE as a distinct clinical condition based on its characteristic annular, pruritic, and polycyclic morphology, highlighting that the classification of EAE remains unclear in recent literature [8, 9]. Overall, these findings suggest that flame figures may be a variable histologic feature that reflects biopsy timing and lesion maturity rather than a defining feature of EAE. This is consistent with our patient's clinical course, where flame figures were present on the first two biopsies, obtained from well-developed lesions, but absent on the third biopsy performed in 2021.

The pathogenesis of EAE remains poorly understood, but it may be associated with IL-5 mediated eosinophil migration in response to an unidentified trigger [2]. Several chronic diseases, malignancies, and infections have been reported in association with EAE, but no clear etiology has been established in most reported cases, including ours [1, 10]. Our patient’s persistent eosinophilia is consistent with previous reports of cases of chronic EAE [10].

There is currently no standardized treatment for EAE or WS. Initial management often includes topical or systemic corticosteroids, as well as trials of antihistamines, dapsone, hydroxychloroquine, nicotinamide, methotrexate, mycophenolate mofetil, and calcineurin inhibitors. Narrowband UVB phototherapy has shown benefit in several cases [3, 11]. More recently, case series and case reports have also described the successful use of biologics targeting type 2 inflammatory pathways, such as mepolizumab, benralizumab, and dupilumab in refractory cases of both WS and EAE [3, 8, 9, 12]. Dupilumab was selected for our patient based on this recent evidence.

Dupilumab is a humanized monoclonal antibody that blocks the IL-4 receptor α subunit, inhibiting IL-4/IL-13 signaling and downstream Th2 inflammation [13]. It is FDA-approved for multiple conditions, including atopic dermatitis, asthma, chronic rhinosinusitis with nasal polyposis, eosinophilic esophagitis, prurigo nodularis, and more recently, chronic spontaneous urticaria, and bullous pemphigoid. Common adverse effects of dupilumab include conjunctivitis, blepharitis, injection-site reactions, facial or neck erythema, arthralgia, and transient, paradoxical blood eosinophilia.

The relationship between IL-4/IL-13 blockade and eosinophilic inflammation is complex. Dupilumab does not directly target IL-5 signaling or eosinophils, and transient or persistent eosinophilia is a well-recognized effect of treatment. For example, dupilumab has been associated with paradoxical reactions such as the development of eosinophilic granulomatosis with polyangiitis in some patients with underlying eosinophilic airway disease, while other reports have described its successful use in treating eosinophilic fasciitis [14, 15].

Both EAE and WS typically follow a chronic, relapsing course, and our patient's own 26-year history has included periods of partial response to multiple agents followed by relapse. In previous reports of EAE managed with dupilumab, patients experienced rapid clinical improvement, with sustained remission during continued treatment [3]. In two reported cases of WS, dupilumab combined with oral prednisone also achieved sustained remission [16, 17]. The clinical response in our patient provides further support for a potential role of IL-4/IL-13 mediated type 2 inflammation in EAE, although a definitive role cannot be established from a single therapeutic response.

Our report has several limitations. The 9-month follow-up period is a short period of time to determine long-term response in a patient with such a longstanding and relapsing disease course. In addition, it is unfortunate that the original histopathology slides from 2000, 2004, and 2021 were unavailable for review. A complete evaluation for secondary causes of eosinophilia was also not performed during the patient's disease course, although the patient’s clinical presentation, serial biopsies, and absence of other clinical symptoms over 26 years strongly support a diagnosis of EAE.

In summary, our case is notable for a 26-year history of persistent disease refractory to multiple therapies, including omalizumab, followed by a rapid and sustained response to dupilumab. Our findings add to the growing evidence that EAE and WS appear to lie on a clinical spectrum, and suggest that dupilumab may represent a promising therapeutic option for refractory chronic EAE.

## Statement of Ethics

Ethical approval is not required for this study in accordance with local or national guidelines. Written informed consent was obtained from the patient for publication of the details of their medical case and any accompanying images.

## Conflict of Interest Statement

The authors have no conflicts of interest to declare.

## Author Contributions

Avery Seward: writing and original draft preparation. Robert Pariser: conceptualization, review of draft, and clinical care.

## Data Availability

All data generated or analyzed during this study are included in this article and its supplementary material files. Further enquiries can be directed to the corresponding author.
